# Development and validation of a questionnaire to examine determinants of consumer intentions to purchase organic food

**DOI:** 10.1186/s40795-023-00731-y

**Published:** 2023-06-26

**Authors:** Marjan Bazhan, Farnam Shafiei Sabet, Nasrin Borumandnia

**Affiliations:** 1 Department of Community Nutrition, Faculty of Nutrition Sciences and Food Technology, National Nutrition and Food Technology Research Institute, Shahid Beheshti University of Medical Sciences, Tehran, IR Iran; 2 Department of Food Science and Technology, Faculty of Nutrition Sciences and Food Technology, National Nutrition and Food Technology Research Institute, Shahid Beheshti University of Medical Sciences, Tehran, Iran; 3 Urology and Nephrology Research Center, Shahid Beheshti University of Medical Sciences, Tehran, Iran

**Keywords:** Organic food, Purchase intention, Questionnaire, Development, Validation

## Abstract

**Background:**

Organic farming is a relatively new concept in developing countries compared to developed countries. Understanding the factors affecting consumers’ willingness to pay for organic foods is critical to increasing the production of these products. This study aimed to develop and validate a Persian version of the questionnaire for assessing determinants of organic food purchase intention among adults in Tehran, the capital of Iran.

**Methods:**

The study was conducted in a two-phased standardized methodology in 2019. During Phase 1, a draft questionnaire was developed based on a comprehensive literature review. In phase 2, validation of the instrument was performed. Participants included a multidisciplinary expert panel comprising 14 members to evaluate content validity, a sample of lay people to assess face validity (n = 20), internal consistency (n = 300), and test-retest reliability (n = 62). The internal consistency and test-retest reliability were measured using the intraclass correlation coefficient (ICC) and Cronbach’s alpha.

**Results:**

Forty-nine of 57 items had a CVR above 0.51 and were retained in the questionnaire. Three items were added to the questionnaire. The average CVI for the questionnaire was 0.97. Cronbach’s α and ICC of the entire questionnaire were 0.86 and 0.93, respectively. Each phase of development progressively improved the questionnaire, resulting in a final 52-item questionnaire divided into 9 dimensions, including knowledge, attitude, subjective norms, health consciousness, environmental concerns, perceived convenience of purchase, perceived cost, sensory characteristics, and purchase intention.

**Conclusions:**

The developed questionnaire appears to be a valid and reliable instrument for examining determinants of consumer intentions to purchase organic food.

**Supplementary Information:**

The online version contains supplementary material available at 10.1186/s40795-023-00731-y.

## Background

Official statistics show that the global sales of organic foods and beverages reached around USD 220.00 Billion in 2019. The market is expected to reach USD 620.00 billion by 2026, with a compound annual growth rate (CAGR) of 16.0% between 2019 and 2026 [1]. Organic foods are produced in about 180 countries of the world. The United States and the European Union ranked as the first and second biggest regions in market share with approximately 42% and 38.5%, respectively [2]. The production and consumption of organic food products were initially more popular in developed countries. Today, however, this concept is also accepted in developing countries, in a way that about one-third of the world’s organically managed land is located in developing countries [3]. Even though the tendency to produce and consume organic foods has increased in various countries to protect the environment and human health, impressive steps have not been taken to plan, guide, and protect organic farming in Iran. According to the global report in 2018, less than 1% of agricultural land in Iran is under organic management [2]. On the other hand, the changes in Iranians’ dietary patterns and nutritional transitions from traditional to fast food intake have been identified as one of the most important social trends affecting the future health system of Iran that requires special attention and actions by policymakers in this field [4].

Consumer demand plays a pivotal role in the future of organic agriculture. Production and marketing strategies are determined by a range of consumer-related factors such as beliefs, attitudes, responses to organically grown products, and the willingness to pay a premium price for them [5–7]. Therefore, it is crucial to understand consumer willingness and decisional factors driving organic food purchase intention [8]. Over the past few years, many studies have extensively assessed issues related to the market of organic food products [9]. The majority aimed to clarify the role of psychological, socio-demographic, and economic factors in consumers’ choices [10–17]. However, various studies have yielded contradictory results. For instance, some researchers believe that altruistic aspects of the concept (e.g., environmental awareness, animal welfare, and fair trade) were the most critical factors affecting the consumption of organic food products [10, 13, 15, 18–21]. Other researchers have reported individual aspects such as awareness of the organic food label, health concerns, nutritional value, food safety, taste, and freshness as the main determinants of organic food consumption [11, 16, 17, 20, 22–27]. The lack of consistency between the results of various studies might be due to differences in sample sizes and the generalizability of the findings, regional focus, type of products being studied, and market sizes for organic foods [27].

Previous studies on organic food purchase intention/ behavior have mostly been done in the United States and the European continent. In contrast, very few studies focused on the consumers’ perception of organic foods in Asia [28]. In particular, there is a lack of studies on consumption trends in South-West Asian countries (i.e., Turkey, the United Arab Emirates, Oman, Qatar, Iran, etc.). It may be because organic farming is a relatively new concept in developing countries compared to developed countries. Given the numerous economic, social, and environmental advantages of organic foods and the potential for expanding the production and consumption of these products, examining various aspects of the organic food purchase intention in developing countries such as Iran is necessary. Identifying the factors affecting consumers’ willingness to pay for organic foods and prioritizing them can provide helpful information for agricultural traders, manufacturers, and policymakers [29] to implement supportive policies and meet the needs of producers and consumers.

There are instruments already developed to assess purchase intention or behavior toward organic foods [30–34]. However, to the best of our knowledge, no previous research has developed a valid instrument to assess the various factors affecting consumers’ intention to purchase organic food in Iran. In a recent study designed to investigate the willingness of Iranian young adults to eat organic foods [35], researchers used a questionnaire developed based on the literature review and the Health Belief Model (HBM). Only some predictors of willingness to use organic foods, solely individual factors, were examined in this study. The present study aimed to develop a valid and wide-ranging questionnaire in the Iranian context to assess these parameters in adults based on an expanded version of the theory of planned behavior (TPB).

## Methods

### Theoretical framework

We used the Theory of Planned Behavior (TPB) as the theoretical framework. The TPB, developed by Fishbein & Ajzen, provides a valuable framework for predicting and explaining health behaviors. According to this theory, the intention to perform a particular behavior is the best predictor of that behavior. Behavioral intention is determined by three factors: attitude toward the behavior, subjective norm concerning the behavior, and perceived behavioral control [36]. The TPB is, in principle, open to including additional predictors [37] to improve the predictive utility of the TPB across various domains [38]. Taking support from the extant literature, we included six constructs (knowledge, health consciousness, environmental concerns, perceived convenience of purchase, perceived cost, and sensory characteristics) in the TPB.

### Study design

**Fig. 1:**
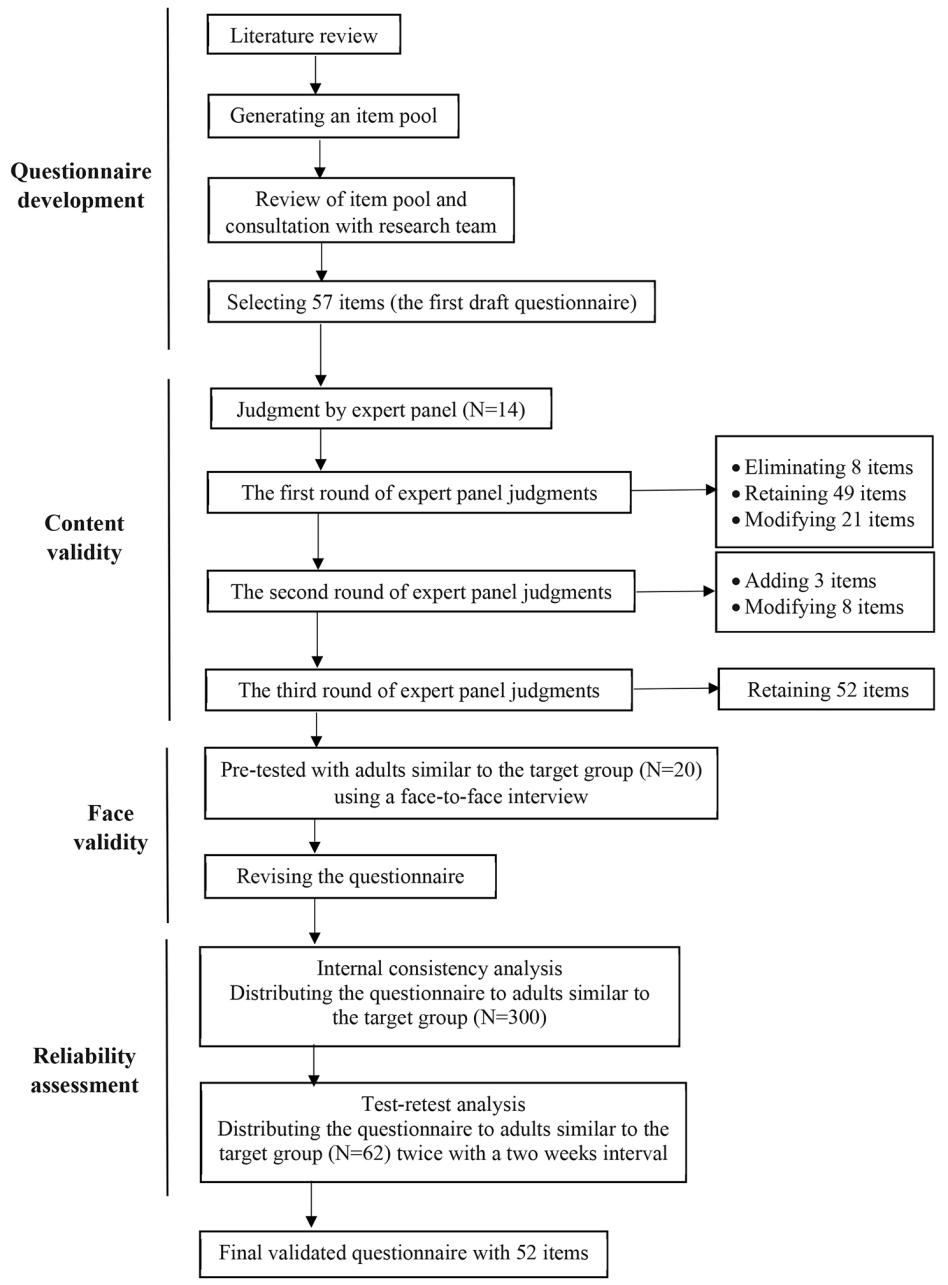
Flow diagram describing the steps followed to develop and validate the questionnaire to examine determinants of consumer intentions to purchase organic food

This cross-sectional methodological study was conducted in Tehran, the capital of Iran, in 2019. It was done in two-phase: (1) questionnaire development in the Persian language, (2) validation in the Iranian context. Figure 1 presents an overview of the questionnaire development process.

### Phase 1: questionnaire development

A comprehensive literature review using search engines like Google Scholar and PubMed was performed to identify publications on the intention or behavior to buy organic foods. Keywords such as “organic food”, “organic products”, “purchase intention”, “intention to buy”, and “behavior” were included in the search. Studies were included for the review if they met the following criteria: [1] focused on the factors affecting the purchasing intention or behavior regarding organic foods [2], published in English or Persian language [3], published between the years 1990 and 2018. Questionnaire items were generated from the selected studies. After removing duplicated or similar items, the initial questionnaire was prepared using 57 items categorized under the nine dimensions mentioned above. The items to examine consumer knowledge had three options: correct, wrong, and I do not know. Other items were rated on a 5-point Likert-type scale ranging from 1 (completely disagree) to 5 (completely agree). Negatively stated items were reverse-scored.

Individual factors must be acknowledged when researching organic foods, as they affect the purchase intention of these products. We developed questions about socio-demographic characteristics to form a pool of supplemental questions. However, they were not included in the validity and reliability assessment.

### Phase 2: validation

- Content Validity: Content validity refers to the degree to which an assessment or measurement tool accurately measures the various aspects of a specific concept [39]. To determine the instrument’s content validity in qualitative and quantitative methods, a panel of 14 multidisciplinary experts related to the research topic (4 nutritionists, 3 food scientists, 3 health education and health promotion specialists, and 4 agricultural specialists), recruited through a purposive sampling technique examined the initial questionnaire. The questionnaire was e-mailed to experts to gather their opinions and comments, with 2 reminder e-mails sent at 2-week intervals. In all, the responses of 14 members were analyzed.

In the qualitative content validity method, the experts’ opinions on the accuracy of Persian grammar, using appropriate words and scoring, and placing items in their proper place, were employed. Items were modified based on the experts’ comments. The content validity ratio (CVR) and the content validity index (CVI) were calculated in the quantitative content validity method. To calculate the CVR, we requested the experts to score each item using a three-point scale “not necessary, useful but not essential, essential”, respectively. The formula for the CVR is CVR = (Ne – N/2)/ (N/2), where Ne is the number of experts indicating an item as “essential” and N is the total number of experts. The value of CVR for each item was determined by Lawshe’s Table [40]. Then, we asked the experts to rate each item based on relevance, clarity, and simplicity on the four-point scale to get the CVI. CVI can be calculated both for item level (I-CVIs) and scale level (S-CVI) [40]. I-CVI is determined by the proportion of exports judging the item as relevant or clear (rating 3 or 4). S-CVI is calculated in two ways: the Universal Agreement (UA) among experts (S-CVI/UA), and the Average CVIs (S-CVI/Ave), the latter being a less conservative method [40]. In this study, S-CVI/Ave was calculated by dividing the sum of the I-CVIs by the total number of items.

The experts’ judgments led to eliminating 8 items and adding 3 items. Finally, the revised questionnaire contained 52 items.

- Face validity: Face validity is the degree to which the instrument appears to measure what it is supposed to calculate [41]. To confirm the face validity of the tool, we recruited a convenience sample of 20 adults aged 25–65 years from a wholesale fruit and vegetable market in the center of Tehran because it was shown that this sample size could sufficiently detect ambiguous items [42]. We asked the participants to complete a paper version of the questionnaire and participate in a face-to-face interview to provide additional feedback and identify what they thought the questionnaire measured. In addition, individual and mean completion times were recorded.

- Reliability: Reliability is the degree to which an assessment tool produces stable and consistent results [43]. We assessed the instrument’s reliability using internal consistency reliability and test-retest procedure. The internal consistency reliability coefficient was evaluated using Cronbach’s alpha for each domain and the questionnaire. For this purpose, the final questionnaire was given to 300 participants aged 25–65 years recruited from the wholesale fruit and vegetable markets in different geographical areas in Tehran. Reproducibility was examined by test-retest over a 2-week period. Because of the principle of anonymity, the samples recruited from the public did not contain any contact information, which made it impossible to retest. Therefore, assuming the intraclass correlation coefficient (ICC) of 0.80, the 95% Confidence Interval [CI] for ICC = 0.20, and with consideration of an attrition rate of 20% [44], we selected a convenience sample of 62 adults aged 25–65 years to complete the questionnaire twice with a two weeks interval. Respondents completing the reliability testing had not been earlier involved with either the content or face validity testing.

### Statistical analysis

The numeric value of CVR was determined by Lawshe Table. According to the panel of 14 experts in the present study, a CVR greater than 0.51 was acceptable [45]. It means that the item was maintained in the instrument. Judgment on CVI was made as follows: If the I-CVI was higher than 79%, the item was appropriate. If it was between 70 and 79%, it needed revision. It was eliminated if it was less than 70% [40]. The S-CVI/Ave values ≥ 0.9 were considered excellent content validity [46]. Cronbach’s alpha values greater than 0.7 were considered acceptable internal consistency [47]. The test-retest reliability was evaluated using the intraclass correlation (ICC), where an ICC equal to or above 0.7 was acceptable [48]. All statistical analysis was performed using SPSS 21.0 (SPSS Inc., Chicago, Illinois, U.S.).

## Results

A total of 382 adults participated in the face validity and reliability assessment. The characteristics of the adults are shown in Table 1.

**Table 1:** Demographic characteristics of adults participating in validity and reliability studies

Characteristics	Face validity n = 20	Internal consistency reliability n = 300	Test-retest reliability n = 62
***Gender***			
Female (%)	55	62.3	64.5
***Age***			
Mean years (SD)	40.2 (10.1)	38.9 (11.2)	38.3 (9.9)
Minimum- Maximum Years	27–60	25–65	25–64
***Education level*** (%)			
Under diploma	10.0	6.3	4.8
Diploma	50.0	26.7	37.2
University	40.0	67.0	58.0
***Districts in the city*** *(%)*			
North	**-**	20.1	**-**
South	**-**	19.8	**-**
Center	100	21.3	100
West	**-**	19.9	**-**
East	**-**	18.9	**-**

### Phase 1: questionnaire development

We developed the initial questionnaire with 57 items following a review of the literature, divided into 9 dimensions: 10 items for assessing the level of consumer knowledge about organic foods [30, 49], 10 items for measuring attitudes towards the products [30, 31, 50], 3 items on subjective norms [51], 9 items on health consciousness [15, 30], 9 items on environmental concerns [30, 52], 5 items about perceived convenience of purchase [30], 5 items related to perceived cost [30], 4 items on sensory characteristics of the product [53], and 2 items related to organic food purchase intention [54].

### Phase 2: validation

- Content Validity: In the first round of judgment, according to the experts’ panel opinion, 8 items were eliminated, 49 items with a CVR above 0.51 retained in the questionnaire, and necessary modifications were applied to 21 items (Table 2). In the second round, the panel members rated the remaining items of the first round based on relevance, clarity, and simplicity on the four-point scale. Then, CVI for each item was calculated. In this round, 8 items were modified based on the qualitative feedback of the experts on clarity, content, and length. Also, 3 items were added to the questionnaire; 2 items to the knowledge dimension and 1 item to the attitude dimension (Table 3). The instrument containing 52 items was sent to the expert panel in the third round. They judged the items’ relevancy, clarity, simplicity, and the need to delete or add items. In this round, all items had a CVI greater than 0.79, which supports retaining all of them in the instrument [40]. The average content validity index for the questionnaire (S-CVI/Ave) was 0.97, indicating an instrument’s excellent content validity [46].

**Table 2:** Results of the content validity ratio (CVR) and the content validity index (CVI) for the questionnaire

Dimension and items	CVR	Decision	CVI
***Knowledge***			
1. Chemical fertilizers and pesticides are used to produce organic foods.	1.00	Retained	1.00
2. Organic foods are not natural products.	0.39	Eliminated	-
3. Genetic modification is used in the production of organic foods.	0.85	Retained	1.00
4. The nutritional value of organic foods is higher than that of conventional foods.	0.85	Retained	1.00
5. Organic foods do not contain preservatives.	1.00	Retained	1.00
6. Human or animal manure is used in organic farming.	0.85	Retained	1.00
7. It is difficult for me to know whether food is organically produced or not.	1.00	Retained	1.00
8. Organic foods taste better than non-organic foods.	1.00	Retained	1.00
9. Organic foods are fresher than conventional foods.	0.23	Eliminated	-
10. Organic farming supports small local farmers.	0.69	Retained	0.92
***Attitude***			
11. Buying organic foods is a good idea.	0.08	Eliminated	-
12. Buying organic foods is logical and wise.	0.85	Retained	0.92
13. The quality of organic foods is better than non-organic foods.	0.85	Retained	1.00
14. Buying organic foods is enjoyable for me.	-0.85	Eliminated	-
15. I trust organic food producers.	1.00	Retained	1.00
16. I am not interested in buying organic food.	0.85	Retained	1.00
17. I am motivated to buy organic foods because of their beneficial properties.	1.00	Retained	1.00
18. I believe that buying organic foods is better than buying non-organic foods.	0.39	Eliminated	-
19. I am going to buy organic foods because of the positive image I have of them.	0.39	Eliminated	-
20. I do not trust the information on organic food labels.	1.00	Retained	1.00
***Subjective norms***			
21. Many people who are important to me in life think that I should buy organic food.	0.69	Retained	0.92
22. Many people who are important to me in life ask me to buy organic foods.	0.85	Retained	1.00
23. The people whose opinions I value prefer not to buy organic food.	0.69	Retained	0.85
***Health Consciousness***			
24. I care about my health.	-0.85	Eliminated	-
25. Non-organic foods are as healthy as organic foods.	0.69	Retained	1.00
26. Organic foods are natural, so they are better for my health.	0.69	Retained	1.00
27. Organic foods are healthier; because they do not contain hormones.	0.85	Retained	1.00
28. Organic foods are healthier; because they do not contain antibiotics.	0.85	Retained	1.00
29. Organic foods are healthier; because they do not contain toxic or chemical residues.	0.85	Retained	1.00
30. I choose food carefully to make sure it is healthy.	0.69	Retained	0.88
31. I think of myself as a health-conscious consumer.	0.69	Retained	0.85
32. I often think about health-related issues.	0.69	Retained	0.85
***Environmental concerns***			
33. The environmental balance is highly vulnerable and can be easily disrupted.	0.85	Retained	0.92
34. Human beings do not use the environment properly.	0.69	Retained	0.92
35. Human beings must maintain the balance of the environment for survival.	0.85	Retained	0.85
36. Improper human interference in the environment can lead to catastrophic consequences.	0.85	Retained	0.92
37. The environment must be protected by using environmentally friendly farming methods.	0.85	Retained	0.92
38. The production of food products in conventional ways does not harm the environment.	1	Retained	1.00
39. Organic foods production is better for the environment; because in this method, pesticides and chemical fertilizers are not used at all or are used in lesser amounts.	0.85	Retained	0.92
40. Organic foods production is better for the environment; because in this method, Hormones are not used at all or are used in lesser amounts.	0.69	Retained	0.85
41. Organic farming methods are better for the environment than conventional methods.	0.85	Retained	1.00
***Perceived convenience of purchase***			
42. Organic foods are available in sufficient quantities in the stores where I do shopping.	1	Retained	1.00
43. It is hard to find organic foods in the stores where I go shopping.	0.23	Eliminated	-
44. If there are organic foods in the stores where I go shopping, I think of buying them.	1	Retained	1.00
45. I can easily find organic foods in my neighborhood.	0.69	Retained	1.00
46. I intend to buy organic foods, provided they are more accessible in the market.	0.69	Retained	1.00
***Perceived price***			
47. The price of organic foods is very important to me.	0.85	Retained	1.00
48. I often refuse to buy organic foods; because I think they are expensive.	0.85	Retained	1.00
49. It is important to me that the price of organic foods be similar to that of non-organic foods.	1	Retained	1.00
50. I always try to find cheap foods while shopping.	0.69	Retained	0.92
51. I intend to buy organic foods, provided they are sold at a lower price.	0.85	Retained	1.00
***Sensory characteristics***			
52. Organic food products taste good.	1	Retained	1.00
53. The appearance of organic foods is not appealing and attractive.	1	Retained	1.00
54. Organic foods have a good and pleasant texture.	1	Retained	1.00
55. Organic foods packaging is not attractive.	1	Retained	1.00
***Purchase intention***			
56. I am willing to buy organic food while shopping.	0.85	Retained	0.92
57. I will make an effort to buy organic food in the near future.	1	Retained	1.00

**Table 3:** Items added to the questionnaire based on the opinions of the expert panel

Dimension	Added item	Reason for adding the item to the questionnaire
Knowledge	Hormones are used in the production of organic foods.	None of the items included in the questionnaire measured these aspects of organic foods.
	Antibiotics are not used in the production of organic foods.	
Attitude	I trust the organic certification mark on the packaging.	

At the end of the third round, the revised questionnaire comprised 52 items in 9 dimensions, including knowledge (10 items), attitude (7 items), subjective norms (3 items), health consciousness (8 items), environmental concerns(9 items), perceived convenience of purchase (4 items), perceived cost (5 items), sensory characteristics (4 items), and organic food purchase intention (2 items).

- Face validity: approximately 92% of participants identified that the survey measured factors affecting consumer intentions to purchase organic food. The time for questionnaire completion, together with the supplemental questions, was 20 ± 5 min. Moreover, 93%of participants reported that the questionnaire length was appropriate. Minor modifications were made to flow and clarity.

- Reliability: The Cronbach’s alpha and intraclass correlation coefficient for all questionnaire dimensions are shown in Table 4. Cronbach’s alpha coefficient ranged from 0.71 to 0.88 for various dimensions. The alpha value for the overall questionnaire was 0.86, indicating appropriate internal consistency reliability [47]. The ICC of the entire questionnaire was 0.93 (ranging from 0.84 to 0.99), revealing satisfactory stability [48].

**Table 4:** Cronbach’s α coefficient and ICC for various dimensions of the questionnaire^*^

The questionnaire dimensions	Cronbach’s α	ICC (%95 CI)
Knowledge	0.78	0.92 (0.84–0.96)
Attitude	0.71	0.85 (0.82–0.93)
Subjective norms	0.74	0.90 (0.79–0.95)
Health consciousness	0.74	0.84 (0.76–0.92)
Environmental concerns	0.75	0.86 (0.72–0.93)
Perceived convenience of purchase	0.76	0.92 (0.84–0.96)
Perceived cost	0.79	0.96 (0.92–0.98)
Sensory Characteristics	0.78	0.91 (0.82–0.96)
Purchase Intention	0.88	0.99 (0.99-1.00)

^*^ ICC = Intraclass Correlation Coefficient; CI = Confidence Interval

## Discussion

The present study developed a valid and reliable instrument to assess determinants of the intention to purchase organic food. This study followed the two-step method, involving instrument design through a comprehensive literature review and examining psychometric properties through content and face validity, internal consistency, and test-retest reliability.

The questionnaire development process involved a literature search, reviewing the findings from existing literature, and highlighting any gaps in the current research. The review highlighted the limited availability of studies reporting the development of validated tools used to assess the determinants of the intention to purchase organic food in South-West Asian countries, such as Iran. Most of the studies in this field have been conducted in European and American countries [28]. To our knowledge, this study was the first attempt to develop and validate a wide-ranging questionnaire to determine the factors affecting purchase intention toward organic food among Iranian adults based on an expanded version of the theory of planned behavior (TPB).

Food choice and eating behaviors are crucial for a healthy lifestyle, with multifactorial determinants rooted in food-related features, individual differences, and society-related features [55]. The same goes for switching to organic eating. Studies have shown that socio-demographic characteristics of the consumer, such as age [56, 57], gender [28, 58], education [56, 57], and income [58, 59], are potential determinants of intention to buy organic food. Other personal factors such as knowledge [34, 60], attitude [3, 61], environmental concerns [62, 63], and health consciousness [3, 61] influence the purchase of organic food. Besides the above, sensory and non-sensory characteristics, including taste [64, 65], appearance [66], freshness [28, 65], package [67], accessibility [21, 68], and price [69, 70], affect the intention to buy these products. The complex and multifaceted nature of individual food choices confirms the necessity to use a multidimensional tool for identifying factors affecting it. The designed questionnaire includes a wide range of items to assess individual and interpersonal factors influencing purchase intention towards organic food. The questionnaire generally showed strong content and face validity, good inter-item reliability, and substantial test-retest reliability.

In this study, the content validity was evaluated by expert specialists. Although a 5–10 expert panel is considered sufficient [71], the study exceeded this expectation. In developing this instrument, the focus was on designing a questionnaire that could be completed independently and without assistance. Therefore, questions needed to be pitched appropriately for the people filling out the questionnaire. Face validity adds further confidence that target populations will find the questionnaire acceptable and understandable.

A literature review related to the present study’s area of inquiry indicated some attempts to test the questionnaire’s psychometric properties but were mostly limited to construct validity and internal consistency reliability [32–35, 72, 73]. Accordingly, it was only possible to compare the findings related to measuring the internal consistency reliability of our tool with other studies whose instrumental dimensions were somewhat similar to the present study.

The reproducibility of most of the items in this questionnaire was similar to those previously designed in other communities. For instance, our results are in agreement with the study of Teng & Wang [32], who developed a questionnaire with six dimensions, four of which were in line with the dimensions of our questionnaire. They tested its validity and reliability to identify decisional factors driving organic food consumption in adults between 18 and 70 years old in Taiwan. Cronbach’s alpha values of their questionnaire ranged from 0.77 to 0.87 [32]. Our findings are also compatible with the 44-item questionnaire developed by Voon et al. [33]. Some dimensions of this tool are almost similar to our questionnaire. Estimating reliability using Cronbach’s alpha showed a satisfactory internal consistency among the dimensions with values between 0.73 and 0.96 [33]. Likewise, in 2017, Singh and Verma [34] validated a 22-item questionnaire in eight dimensions to identify factors influencing Indian consumers’ purchase intentions and actual purchase behavior of organic foods. The dimensions Cronbach’s alpha ranged from 0.77 to 0.89 [34], which accords with the results of our study. A 29-item tool was developed for examining the factors affecting organic food purchase intention among Malaysian consumers. Among the dimensions of this questionnaire, five dimensions are nearly consistent with our questionnaire. Cronbach’s alpha values for all dimensions surpassed the acceptable value of 0.70 [73]. In 2019, evaluating the reliability of the questionnaire developed to assess the relationship between trust factors and buying behavior toward organic food in Taiwan using Cronbach’s alpha coefficient showed a high internal consistency (alpha = 0.94) [72], higher than the present study’s result. The lower coefficients of some dimensions in our questionnaire compared to those designed in other communities might be because we used a mixture of positive and negative items. However, the pilot study revealed that the questionnaire would be more reliable if we used only positive words.

Demographic questions were purposively inserted at the end of the questionnaire, as it has been suggested that these questions can be considered threatening [74] or boring [75].

The strength of the present study lies in its rigorous examination of validity and reliability. Unlike the previous research [32–35, 72], this study examined the psychometric properties in several methods, including content and face validity, internal consistency, and test-retest reliability. Input from a range of experts, besides a high CVI (0.80), builds confidence that this questionnaire accurately and reliably assesses organic food purchase intention. The other strength is developing a multidimensional tool to assess determinants of behavioral intention on organic foods. The assessment of different factors within the same measurement tool makes it possible to compare them simultaneously, identify each dimension’s relative importance, and prioritize them. Our study has some limitations. First, the questionnaire developed in this study comprises many questions. Questionnaires with a long list of questions may negatively affect the participation rate and the quality of data [76]. However, most participants and experts evaluating the questionnaire found it appropriate. Second, this questionnaire has been designed in the Persian language and validated in a sample of Iranian subjects. Considering that consumers’ purchase intentions and choices are influenced by culture and society, a cross-cultural adaption of the questionnaire should be undertaken before submitting it to other cultures [77] and re-examined for validity and reliability. At present, Persian and English versions of the questionnaire (excluding questions about socio-demographic characteristics) are available (S1 and S2 files).

## Conclusions

The present study developed a valid and reliable questionnaire (self-complete) instrument to examine the various factors affecting the consumers’ intention to purchase organic foods. Future studies could apply our tool to examine their population of interest and use the results to leverage the facilitators and limit the barriers in their methodologies when designing interventions to promote organic food consumption. The generalizability and, therefore, implications are limited to the Iranian population.

## Electronic supplementary material

Below is the link to the electronic supplementary material.

Supplementary Material 1

Supplementary Material 2

## Data Availability

The datasets used and/or analyzed during the current study are available from the corresponding author on reasonable request.

## References

[1] Khangan M. Organic Food And Beverages Market Size to Reach USD 620.00 Billion by 2026, Globally. Food & Beverages. 2020:1-220. https://orgprints.org/id/eprint/38396/1/Organic%20Food%20And%20Beverages%20Market%20Size%20to%20Reach%20USD%20620.00%20Billion%20by%202026,%20Globally.pdf

[2] Willer H, Sahota A. The world of organic agriculture, statistics and emerging trends 2020 at BIOFACH 2020. BIOFACH Congress 2020; Messezentrum Nürnberg, Germany12–15 February 2020. https://orgprints.org/id/eprint/37557/1/willer-et-al-2020-global-stats.pdf

[3] Yadav R, Pathak GS (2016). Intention to purchase organic food among young consumers: evidences from a developing nation. Appetite.

[4] Rahimi H, Kalantari A, Rafiee N, Khosravi S (2019). Social trends affecting the future of Iran’s health system: a qualitative study using focus group discussion. Int J Prev Med.

[5] Aryal KP, Chaudhary P, Pandit S, Sharma G (2009). Consumers’ willingness to pay for organic products: a case from Kathmandu valley. J Agric Environ.

[6] Parker JS, Wilson RS, LeJeune JT, Rivers L, Doohan D (2012). An expert guide to understanding grower decisions related to fresh fruit and vegetable contamination prevention and control. Food Control.

[7] Rembiałkowska E (2007). Quality of plant products from organic agriculture. J Sci Food Agric.

[8] Eyinade GA, Mushunje A, Yusuf SFG (2021). The willingness to consume organic food: a review. Food and Agricultural Immunology.

[9] Gracia A, de Magistris T (2008). The demand for organic foods in the South of Italy: a discrete choice model. Food Policy.

[10] Michaelidou N, Hassan LM (2010). Modeling the factors affecting rural consumers’ purchase of organic and free-range produce: a case study of consumers’ from the island of Arran in Scotland, UK. Food Policy.

[11] Haghiri M, Hobbs JE, McNamara ML (2009). Assessing consumer preferences for organically grown fresh fruit and vegetables in Eastern New Brunswick. Editorial Staff.

[12] de Magistris T, Gracia A (2008). Factors influencing organic food purchase in India–expert survey insights. Br Food J.

[13] Chen M-F (2007). Consumer attitudes and purchase intentions in relation to organic foods in Taiwan: moderating effects of food-related personality traits. Food Qual Prefer.

[14] Honkanen P, Verplanken B, Olsen SO (2006). Ethical values and motives driving organic food choice. J Consumer Behav.

[15] Tarkiainen A, Sundqvist S (2005). Subjective norms, attitudes and intentions of finnish consumers in buying organic food. Br food J.

[16] Magnusson MK, Arvola A, Hursti U-KK, Åberg L, Sjödén P-O (2003). Choice of organic foods is related to perceived consequences for human health and to environmentally friendly behaviour. Appetite.

[17] Zanoli R, Naspetti S (2002). Consumer motivations in the purchase of organic food: a means-end approach. Br food J.

[18] McEachern MG, Willock J (2004). Producers and consumers of organic meat: a focus on attitudes and motivations. Br Food J.

[19] Kareklas I, Carlson JR, Muehling DD (2014). I eat organic for my benefit and yours”: egoistic and altruistic considerations for purchasing organic food and their implications for advertising strategists. J Advertising.

[20] Çabuk S, Tanrikulu C, Gelibolu L (2014). Understanding organic food consumption: attitude as a mediator. Int J consumer Stud.

[21] Lee H-J (2016). Individual and situational determinants of US consumers’ buying behavior of organic foods. J Int Food Agribusiness Mark.

[22] Chen M-F (2009). Attitude toward organic foods among taiwanese as related to health consciousness, environmental attitudes, and the mediating effects of a healthy lifestyle. Br Food J.

[23] Makatouni A (2002). What motivates consumers to buy organic food in the UK? Results from a qualitative study. Br Food J.

[24] McEachern MG, Mcclean P (2002). Organic purchasing motivations and attitudes: are they ethical?. Int J Consumer Stud.

[25] Mondelaers K, Verbeke W, Van Huylenbroeck G (2009). Importance of health and environment as quality traits in the buying decision of organic products. Br Food J.

[26] Padel S, Foster C (2005). Exploring the gap between attitudes and behaviour: understanding why consumers buy or do not buy organic food. Br food J.

[27] Padilla Bravo C, Cordts A, Schulze B, Spiller A (2013). Assessing determinants of organic food consumption using data from the German National Nutrition Survey II. Food Qual Prefer.

[28] Roitner-Schobesberger B, Darnhofer I, Somsook S, Vogl CR (2008). Consumer perceptions of organic foods in Bangkok, Thailand. Food Policy.

[29] Van Loo EJ, Caputo V, Nayga RM, Meullenet J-F, Ricke SC (2011). Consumers’ willingness to pay for organic chicken breast: evidence from choice experiment. Food Qual Prefer.

[30] Olivová K. Intention to buy organic food among consumers in the Czech Republic. Master’s Thesis, University of Agder, Kristiansand, Norway, 2011. https://uia.brage.unit.no/uia-xmlui/bitstream/handle/11250/135628/BE-501%202011%20spring%20Master%20thesis%20Kristyna%20Olivova.pdf?sequence=1

[31] Tsakiridou E, Boutsouki C, Zotos Y, Mattas K (2008). Attitudes and behaviour towards organic products: an exploratory study. Int J Retail Distribution Manage.

[32] Teng C-C, Wang Y-M (2015). Decisional factors driving organic food consumption. Br Food J.

[33] Voon TJP, Ngui KS, Agrawal A. Determinants of willingness to purchase organic food: an exploratory study using structural equation modeling. Int Food Agribusiness Manage Rev. 2011;14. 10.22004/ag.econ.103989. (1030-2016-82772):103 – 20.

[34] Singh A, Verma P (2017). Factors influencing indian consumers’ actual buying behaviour towards organic food products. J Clean Prod.

[35] Yazdanpanah M, Forouzani M, Hojjati M (2015). Willingness of iranian young adults to eat organic foods: application of the Health Belief Model. Food Qual Prefer.

[36] Ajzen I (1991). The theory of planned behavior. Organ Behav Hum Decis Process.

[37] Ajzen I (2020). The theory of planned behavior: frequently asked questions. Hum Behav Emerg Technol.

[38] Donald IJ, Cooper SR, Conchie S (2014). An extended theory of planned behaviour model of the psychological factors affecting commuters’ transport mode use. J Environ Psychol.

[39] Heale R, Twycross A (2015). Validity and reliability in quantitative studies. Evid Based Nurs.

[40] Polit DF, Beck CT, Owen SV (2007). Is the CVI an acceptable indicator of content validity? Appraisal and recommendations. Res Nurs Health.

[41] Cottrell RR, McKenzie JF. Health promotion and education research methods: Using the five-chapter thesis/dissertation model: Jones & Bartlett Publishers; 2010.

[42] Streiner DL, Norman GR, Cairney J. Health measurement scales: a practical guide to their development and use. Oxford University Press, USA; 2015.

[43] Kimberlin CL, Winterstein AG (2008). Validity and reliability of measurement instruments used in research. Am J health-system Pharm.

[44] Liu TW, Lam SC, Chung MH, Ho KHM. Adaptation and psychometric testing of the hoarding rating scale (HRS): a self-administered screening scale for epidemiological study in Chinese population. BMC psychiatry. 2020;20(1):1–10. 10.1186/s12888-020-02539-7 10.1186/s12888-020-02539-7 PMC7155259 32290825

[45] Polit D, Yang F (2016). Measurement and the measurement of change: a primer for the Health Professions.

[46] Shi J, Mo X, Sun Z (2012). Content validity index in scale development. Zhong nan da xue xue bao Yi xue ban = Journal of Central South. Univ Med Sci.

[47] Tavakol M, Dennick R (2011). Making sense of Cronbach’s alpha. Int J Med Educ.

[48] Polit D, Beck C (2017). Nursing research: Generating and assessing evidence for nursing practice.

[49] Yi LK (2009). Consumer behaviour towards organic food consumption in Hong Kong: an empirical study.

[50] Wang Y, Wiegerinck V, Krikke H, Zhang H (2013). Understanding the purchase intention towards remanufactured product in closed-loop supply chains: an empirical study in China. Int J Phys Distribution Logistics Manage.

[51] Mhlophe B (2016). Consumer purchase intentions towards organic food: insights from South Africa. Bus Social Sci J.

[52] Roberts JA, Bacon DR (1997). Exploring the subtle relationships between environmental concern and ecologically conscious consumer behavior. J Bus Res.

[53] Steptoe A, Pollard TM, Wardle J (1995). Development of a measure of the motives underlying the selection of food: the food choice questionnaire. Appetite.

[54] Lee J-S, Hsu L-T, Han H, Kim Y (2010). Understanding how consumers view green hotels: how a hotel’s green image can influence behavioural intentions. J sustainable tourism.

[55] Chen P-J, Antonelli M (2020). Conceptual models of Food Choice: influential factors related to Foods, individual differences, and Society. Foods.

[56] Bravo CP, Cordts A, Schulze B, Spiller A (2013). Assessing determinants of organic food consumption using data from the German National Nutrition Survey II. Food Qual Prefer.

[57] Siegrist M, Hartmann C (2019). Impact of sustainability perception on consumption of organic meat and meat substitutes. Appetite.

[58] Rimal AP, Moon W, Balasubramanian S (2005). Agro-biotechnology and organic food purchase in the United Kingdom. Br Food J.

[59] Cranfield JA, Magnusson E (2003). Canadian consumers’ willingness to pay for pesticide-free food products: an ordered probit analysis. Int Food Agribusiness Manage Rev.

[60] Andervazh L (2020). Jalili s, zanjani s. studying the factors affecting the attitude and intention of Buying Organic food consumers: structural equation model. Iran J Health Educ Health Promotion.

[61] Wang X, Pacho F, Liu J, Kajungiro R (2019). Factors influencing organic food purchase intention in developing countries and the moderating role of knowledge. Sustainability.

[62] Smith S, Paladino A (2010). Eating clean and green? Investigating consumer motivations towards the purchase of organic food. Australasian Mark J (AMJ).

[63] Pagiaslis A, Krontalis AK (2014). Green consumption behavior antecedents: environmental concern, knowledge, and beliefs. Psychol Mark.

[64] Aertsens J, Mondelaers K, Verbeke W, Buysse J, Van Huylenbroeck G (2011). The influence of subjective and objective knowledge on attitude, motivations and consumption of organic food. Br Food J.

[65] Sangkumchaliang P, Huang W-C (2012). Consumers’ perceptions and attitudes of organic food products in Northern Thailand. Int Food Agribusiness Manage Rev.

[66] Ghorbani M, Hamraz S (2009). A survey on factors affecting on consumer’s potential willingness to pay for organic products in Iran (a case study). Trends in Agricultural Economics.

[67] Ibitoye O, Nawi NM, Kamarulzaman NH, Man N (2014). Consumers’ awareness towards organic rice in Malaysia. Int Food Res J.

[68] Hossain M, Lim P (2016). Consumers’ buying behavior towards Organic Foods: evidence from the Emerging Market. Malaysian Manage Rev.

[69] Aertsens J, Verbeke W, Mondelaers K, Van Huylenbroeck G (2009). Personal determinants of organic food consumption: a review. Br Food J.

[70] Nguyen HV, Nguyen N, Nguyen BK, Lobo A, Vu PA (2019). Organic food purchases in an emerging market: the influence of consumers’ personal factors and green marketing practices of food stores. Int J Environ Res Public Health.

[71] Gilbert GE, Prion S (2016). Making sense of methods and measurement: Lawshe’s content Validity Index. Clin Simul Nurs.

[72] Lee TH, Fu C-J, Chen YY (2020). Trust factors for organic foods: consumer buying behavior. Br Food J.

[73] Saleki R, Quoquab F, Mohammad J (2019). What drives malaysian consumers’ organic food purchase intention? The role of moral norm, self-identity, environmental concern and price consciousness. J Agribusiness Developing Emerg Economies.

[74] Lor M, Bowers B, Krupp A, Jacobson N. Ailored explanation: a strategy to minimize nonresponse in demographic items among low-income racial and ethnic minorities. Surv Pract. 2017 10(3). 10.29115/SP-2017-0015 PMC5844486 29527423

[75] Rattray J, Jones MC (2007). Essential elements of questionnaire design and development. J Clin Nurs.

[76] Sharma H (2022). How short or long should be a questionnaire for any research? Researchers dilemma in deciding the appropriate questionnaire length. Saudi J Anaesth.

[77] Beaton DE, Bombardier C, Guillemin F, Ferraz MB (2000). Guidelines for the process of cross-cultural adaptation of self-report measures. Spine.

