# Supplementary material for: Development and validation of a questionnaire to examine determinants of consumer intentions to purchase organic food
Source: BMC Nutr. 2023 Jun 26;9:74. doi: 10.1186/s40795-023-00731-y (PMC10291792; doi:10.1186/s40795-023-00731-y)
Supplement: Supplementary file 1 — Supplementary Material 1 [file 40795_2023_731_MOESM1_ESM.pdf]

پرسشنامه سنجش تعیین‌کننده‌های قصد خرید محصولات غذایی ارگانیک

۱) در جدول زیر فهرستی از جملات آمده است. لطفا نظر خود را در باره هر جمله بیان کنید.

	صحیح	غلط	نمی‌دانم
**سطح دانش**<br>۱- در تولید محصولات غذایی ارگانیک از کودها و آفت‌کش‌های شیمیایی استفاده می‌شود.			
۲- در تولید محصولات غذایی ارگانیک از هورمون استفاده می‌شود.			
۳- در تولید محصولات غذایی ارگانیک، اصلاح ژنتیکی صورت می‌گیرد.			
۴- در تولید محصولات غذایی ارگانیک از آنتی‌بیوتیک استفاده نمی‌شود.			
۵- ارزش غذایی محصولات غذایی ارگانیک نسبت به محصولات غیر ارگانیک بیشتر است.			
۶- در محصولات غذایی ارگانیک، مواد نگهدارنده وجود ندارد.			
۷- درکشاورزی ارگانیک از کود حیوانی یا انسانی استفاده می‌شود.			
۸- برای من سخت است که تشخیص دهم یک ماده غذایی به روش ارگانیک تولید شده یا خیر.			
۹- محصولات غذایی ارگانیک طعم بهتری نسبت به محصولات غیر ارگانیک دارند.			
۱۰- کشاورزی ارگانیک از کشاورزان کوچک محلی حمایت می‌کند.			

۲) در جدول زیر فهرستی از جملات آمده است. لطفاً نشان دهید چقدر با جملات زیر موافق یا مخالف هستید.

	کاملاً موافقم	تاحدی موافقم	نه موافقم نه مخالف	تاحدی مخالفم	کاملاً مخالفم
**نگرش نسبت به محصولات غذایی ارگانیک**<br>۱۱- خرید محصولات غذایی ارگانیک منطقی و عاقلانه است.					
۱۲- کیفیت محصولات غذایی ارگانیک نسبت به محصولات غیر ارگانیک بهتر است.					
۱۳- من به تولیدکنندگان محصولات غذایی ارگانیک اعتماد دارم.					
۱۴- من علاقه‌ای به خرید محصولات غذایی ارگانیک ندارم.					
۱۵- من به علامت گواهینامه ارگانیک روی محصولات غذایی ارگانیک اطمینان دارم.					
۱۶- من به خاطر خواص مفید محصولات غذایی ارگانیک، برای خرید آنها انگیزه دارم.					
۱۷- من به اطلاعات روی برچسب محصولات غذایی ارگانیک اعتماد ندارم.					

**هنجارهای ذهنی**<br>۱۸- بیشتر افرادی که در زندگی برایم مهم هستند، فکر می‌کنند که من باید محصولات غذایی ارگانیک بخرم.					
۱۹- بیشتر افرادی که در زندگی برایم مهم هستند، من را به خرید محصولات غذایی ارگانیک تشویق می‌کنند.					
۲۰- افرادی که نظرشان برایم با ارزش است، ترجیح می‌دهند که من محصولات غذایی ارگانیک نخرم.					
**آگاهی سلامت**<br>۲۱- محصولات غذایی غیرارگانیک به اندازه محصولات ارگانیک سالم هستند.					
۲۲- محصولات غذایی ارگانیک طبیعی هستند، بنابراین برای سلامت من بهتر هستند.					
۲۳- محصولات غذایی ارگانیک سالم‌تر هستند؛ زیرا هورمون ندارند.					
۲۴- محصولات غذایی ارگانیک سالم‌تر هستند؛ زیرا آنتی بیوتیک ندارند.					
۲۵- محصولات غذایی ارگانیک سالم‌تر هستند؛ زیرا باقیمانده‌های سمی و شیمیایی ندارند.					
۲۶- من خودم را یک مصرف‌کنندهٔ دارای آگاهی از مسائل مرتبط با سلامت می دانم.					
۲۷- من غذا را با دقت انتخاب می‌کنم تا از سلامت آن مطمئن شوم.					
۲۸- من اغلب به مسائل مرتبط با سلامت فکر می‌کنم.					
**نگرانی‌های زیست محیطی**<br>۲۹- تعادل محیط زیست بسیار حساس است و می‌تواند به راحتی به هم بریزد.					
۳۰- انسان‌ها از محیط زیست به درستی استفاده نمی‌کنند.					
۳۱- انسان‌ها برای بقای خود باید تعادل محیط زیست را حفظ کنند.					
۳۲- دخالت نابجای انسان در محیط زیست می‌تواند باعث بروز نتایج فاجعه‌بار شود.					
۳۳- با استفاده از روش‌های کشاورزی سازگار با محیط زیست، باید از محیط زیست محافظت کرد.					
۳۴- تولید محصولات غذایی به روش‌هایی که در حال حاضر رایج است، به محیط زیست آسیب نمی‌رساند.					
۳۵- تولید محصولات غذایی ارگانیک برای محیط زیست بهتر است؛ چون در این روش از سم و کود شیمیایی استفاده نمی شود، یا کمتر استفاده می‌شود.					
۳۶- تولید محصولات غذایی ارگانیک برای محیط زیست بهتر است؛ چون در این روش از هورمون استفاده نمی‌شود، یا کمتر استفاده می‌شود.					

۳۷- روش‌های تولید محصولات غذایی ارگانیک نسبت به روش‌های رایج کشاورزی، برای محیط زیست بهتر است.					
**سهولت خرید درک شده**<br>۳۸- محصولات غذایی ارگانیک در فروشگاه‌هایی که من از آنها خرید می‌کنم، به میزان کافی موجود است.					
۳۹- من می‌توانم به راحتی محصولات غذایی ارگانیک را در محله خود پیدا کنم.					
۴۰- اگر محصولات غذایی ارگانیک در جاهایی که خرید می‌کنم وجود داشته باشند، در باره خرید آنها فکر می‌کنم.					
۴۱- من قصد خرید محصولات غذایی ارگانیک را دارم، به شرط اینکه در بازار بیشتر در دسترس باشند.					
**قیمت درک شده**<br>۴۲- قیمت محصولات غذایی ارگانیک برای من بسیار مهم است.					
۴۳- من اغلب از خرید محصولات غذایی ارگانیک خودداری می‌کنم؛ زیرا فکر می‌کنم گران هستند.					
۴۴- برای من مهم است که قیمت محصولات غذایی ارگانیک مشابه محصولات غیر ارگانیک باشد.					
۴۵- من همیشه سعی می‌کنم به هنگام خرید، مواد غذایی با قیمت ارزان را پیدا کنم.					
۴۶- من قصد خرید محصولات غذایی ارگانیک را دارم، به شرط اینکه با قیمت ارزان‌تر فروخته شوند.					
**ویژگی حسی محصول**<br>۴۷- محصولات غذایی ارگانیک طعم و مزه خوبی دارند.					
۴۸- ظاهر محصولات غذایی ارگانیک زیبا و جذاب نیست.					
۴۹- محصولات غذایی ارگانیک بافت خوب و دلپذیری دارند.					
۵۰- بسته بندی محصولات غذایی ارگانیک جذابیتی ندارد.					
**قصد خرید**<br>۵۱- من تمایل دارم به هنگام خرید، محصولات غذایی ارگانیک بخرم.					
۵۲- من تلاش خواهم کرد در آینده نزدیک، محصولات غذایی ارگانیک بخرم.					

© Bazhan et al., 2019
