# Supplementary material for: Development and validation of a questionnaire to examine determinants of consumer intentions to purchase organic food
Source: BMC Nutr. 2023 Jun 26;9:74. doi: 10.1186/s40795-023-00731-y (PMC10291792; doi:10.1186/s40795-023-00731-y)
Supplement: Supplementary file 2 — Supplementary Material 2 [file 40795_2023_731_MOESM2_ESM.pdf]

The questionnaire for assessing determinants of organic foods purchase intention

Section A- Please answer the following questions about organic foods according to your knowledge. Please put a tick in the corresponding box.

Items	True	False	Do not know
1. Chemical fertilizers and pesticides are utilized to produce organic foods.			
2. Hormones are used in the production of organic foods.			
3. Genetic modification is used in the production of organic foods.			
4. Antibiotics are not used in the production of organic foods.			
5. The nutritional value of organic foods is higher than that of conventional foods.			
6. Organic foods do not contain preservatives.			
7. Human or animal manure is used in organic farming.			
8. It is difficult for me to know whether food is organically produced or not.			
9. Organic foods taste better than non-organic foods.			
10. Organic farming supports small local farmers.			

Section B- Please indicate your agreeableness with the sentences on a five-point scale ranging from completely agree to completely disagree. Please put a tick in the corresponding box.

Items	completely agree	agree	No opinion	disagree	completely disagree
**Attitude**<br>11. Buying organic foods is logical and wise.					
12. The quality of organic foods is better than non-organic foods.					
13. I trust organic food producers					
14. I am not interested in buying organic foods.					
15. I trust the organic certification mark on the packaging.					
16. I am motivated to buy organic foods because of their beneficial properties.					
17. I do not trust the information on organic food labels.					
**Subjective norms**<br>18. Many people who are important to me in life think that I should buy organic foods.					
19. Many people who are important to me in life ask me to buy organic foods.					
20. The people whose opinions I value prefer not to buy organic food.					
**Health Consciousness**<br>21. Non-organic foods are as healthy as organic foods.					
22. Organic foods are natural, so they are better for my health.					
23. Organic foods are healthier; because they do not contain hormones.					

24. Organic foods are healthier; because they do not contain antibiotics.					
25. Organic foods are healthier; because they do not contain toxic or chemical residues.					
26. I think of myself as a health-conscious consumer.					
27. I choose food carefully to make sure it is healthy.					
28. I often think about health-related issues.					
**Environmental concerns**<br>29. The environmental balance is highly vulnerable and can be easily disrupted.					
30. Human beings do not use the environment properly.					
31. Human beings must maintain the balance of the environment for survival.					
32. Improper human interference in the environment can lead to catastrophic consequences.					
33. The environment must be protected by using environmentally friendly farming methods.					
34. The production of food products in conventional ways does not harm the environment.					
35. Organic foods production is better for the environment because pesticides and chemical fertilizers are not used at all in this method or are used in lesser amounts.					
36. Organic foods production is better for the environment because hormones are not used at all in this method or are used in lesser amounts.					
37. Organic farming methods are better for the environment than conventional methods.					
**Perceived convenience of purchase**<br>38. Organic foods are available in sufficient quantities in the stores where I do shopping.					
39. I can easily find organic foods in my neighborhood.					
40. If there are organic foods in the stores where I go shopping, I think of buying them.					
41. I intend to buy organic foods, provided they are more accessible in the market.					
**Perceived price**<br>42. The price of organic foods is very important to me.					
43. I often refuse to buy organic foods; because I think they are expensive.					

44. It is important to me that the price of organic foods be similar to that of non-organic foods.					
45. I always try to find cheap foods while shopping.					
46. I intend to buy organic foods, provided they are sold at a lower price.					
**Sensory characteristics**<br>47. Organic food products taste good.					
48. The appearance of organic foods is not appealing and attractive.					
49. Organic foods have a good and pleasant texture.					
50. Organic foods packaging is not attractive.					
**Purchase intention**<br>51. I am willing to buy organic foods while shopping.					
52. I will make an effort to buy organic foods in the near future.					

© Bazhan et al., 2019
